# Examining the associations between linoleic acid‐derived cytochrome P450‐soluble epoxide hydrolase metabolites and small vessel disease markers in normoglycemia, prediabetes, and type 2 diabetes mellitus

**DOI:** 10.1002/alz.095346

**Published:** 2025-01-09

**Authors:** Si Won Ryoo, Myuri Ruthirakuhan, Yuen Yan Wong, William Z. Lin, Natasha Z. Anita, Anthony E Lang, Sean Symons, Stephen R. Arnott, Malcolm Binns, Elizabeth Finger, Sandra E Black, Walter Swardfager

**Affiliations:** ^1^ University of Toronto, Toronto, ON Canada; ^2^ Sunnybrook Research Institute, Toronto, ON Canada; ^3^ Rossy PSP Program, University Health Network and the University of Toronto, Toronto, ON Canada; ^4^ Sunnybrook Health Sciences Centre, Toronto, ON Canada; ^5^ Indoc Systems, Toronto, ON Canada; ^6^ Baycrest Academy for Research and Education, Toronto, ON Canada; ^7^ University of Western Ontario, London, ON Canada; ^8^ Heart and Stroke Foundation Canadian Partnership for Stroke Recovery, Toronto, ON Canada; ^9^ Toronto Dementia Research Alliance, Toronto, ON Canada; ^10^ KITE Toronto Rehabilitation Institute, Toronto, ON Canada

## Abstract

**Background:**

Polyunsaturated fatty acids are metabolized by cytochrome P450 (CYP450) into anti‐inflammatory, pro‐resolving epoxides, which are rapidly converted to inactive and cytotoxic diols by soluble epoxide hydrolase (sEH). Increased CYP450‐sEH metabolites are associated with worse cognition in type 2 diabetes mellitus (T2DM), and greater white matter hyperintensities (WMH) in patients with stroke. We examined whether the relationship between linoleic acid (LA)‐derived CYP450‐sEH metabolites (oxylipins) and small vessel disease (SVD) markers differ across diabetes status.

**Method:**

Cognitively impaired patients with neurodegenerative/ vascular cognitive disorders from the Ontario Neurodegenerative Disease Research Initiative (https://braininstitute.ca/ondri) were classified as having normoglycemia, prediabetes, or T2DM based on a self‐report of diabetes diagnosis, glycated hemoglobin (HbA1c), and antidiabetic medication use. Unesterified plasma oxylipins were quantified via ultra‐high‐performance liquid chromatography tandem mass spectrometry, from which diol to epoxide ratios, a proxy of sEH activity, were calculated. SVD markers included WMH, perivascular spaces (PVS), and lacunes (LACN) quantified through T1‐ and T2‐weighted structural MRI. Linear regression models controlling for age, sex, BMI, intracranial volume, hypertension, *APOE‐ε4* status, HDL, HbA1c, neurodegenerative diagnoses, and antidiabetic medication use, were used.

**Result:**

Among 493 participants, 238 normoglycemic (48.4% female, age = 67.6±8.5 years), 161 prediabetes (35.4% female, age = 69.8±6.8 years), and 94 T2DM participants (20.2% female, age = 69.5±7.2 years) were identified. In the whole group, increased 9,10‐LA ratio was associated with greater PVS (β = 0.097, p = 0.030), but not WMH or LACN. No association was observed with the 12,13‐LA ratio. Significant interaction with HbA1c predicting WMH was observed with the 9,10‐LA ratio (β = 0.530, p = 0.020), and similar effect size was seen with the 12,13‐LA ratio (β = 0.482, p = 0.069). Subgroup analyses revealed a positive association in T2DM only (9,10‐LA: β = 0.313, p = 0.001; 12,13‐LA: β = 0.226, p = 0.020). No interaction effects with HbA1c predicting PVS or LACN were observed. In the normoglycemic subgroup, the 12,13‐LA ratio was negatively associated with LACN (β = ‐0.121, p = 0.025), whereas in T2DM, a positive association was observed (β = 0.213, p = 0.039). Similarly with the 9,10‐LA ratio, in the normoglycemic subgroup, a non‐significant negative association was observed (β = ‐0.100, p = 0.071), whereas in T2DM, a positive association was observed (β = 0.225, p = 0.032).

**Conclusion:**

Diabetes status affects the association between LA‐derived oxylipins and SVD markers. sEH may be a potential therapeutic target in T2DM to reduce neurovascular damage and subsequent cognitive decline.

